# Chronic Low-grade Inflammation after Exercise: Controversies

**Published:** 2012

**Authors:** Majid Khazaei

In a recent published paper in IJBMS, Kazeem A, *et al* evaluated the changes of acute phase proteins after moderate and prolonged exercise (1). They found that prolonged exercise increased plasma C-reactive protein (CRP); however, moderate exercise did not change plasma CRP level. They concluded that moderate exercise, and not prolonged exercise, should be encouraged.

Chronic low-grade inflammation may contribute to pathogenesis of some diseases including cardiovascular disease, diabetes and metabolic syndrome (2). Among inflammatory markers, CRP is the most clinically useful and the best markers of inflammation and is considered as valuable predictors of cardiovascular risk (3). 

The beneficial effects of exercise on cardiovascular system have been documented in experimental and clinical studies by modification of numerous known risk factors (4), however, the exact mechanisms of these protective effects are not fully understood. One of the suggested mechanisms for beneficial effects of exercise in coronary artery disease (CAD) patients is the impact of exercise on vascular inflammation; however, the results are contrary. Some studies reported that exercise is not associated with reduced inflammatory markers (5, 6), while, other studies supported that exercise reduces (7, 8) or even increases those markers (1). This discrepancy depends on some variables including: 

1- Acute vs. chronic exercise: Inflammatory cytokines including IL-6 are produced and released by skeletal muscles. IL-6 is the first inflammatory cytokines released from exercising muscles (9). Therefore, it is clear that acute bout or short-term exercise elevates plasma IL-6 and CRP concentrations and it is related to duration, intensity and muscle mass involved during exercise. A single session of exercise not only increases inflammatory cytokines, but also, elevates oxidative stress and leukocytosis. However, after regular exercise, reduced inflammatory markers and simultaneously increased anti-inflammatory substances are reported (7, 8). 

2- Exercise with or without weight loss: One of the suggested mechanisms for reduced plasma levels of CRP is the decrease in level of adipocyte tissue. Adipocytes are the major sources of inflammatory cytokines such as TNFα and IL-6 (10). It is indicated that exercise training reduced plasma CRP levels in CAD patients regardless of being with or without metabolic syndrome, drug therapy or weight gain or loss (2, 11) and they showed that effect of exercise on CRP is independent of weight loss and statin therapy.

3- Coexistence of chronic diseases: baseline inflammatory markers are important in the effect of exercise. In a large study, 20 weeks of training could not significantly reduce markers in control subjects; however, they showed that in basal high-level CRP, training significantly reduced CRP level (3). On the other hand, although, acute bout of exercise increases serum inflammatory and endothelial 

dysfunction markers, however, in patients with chronic heart failure who have increased level of inflammatory markers, these effects were not observed (12). It is indicated that short-term exercise (2 weeks) in control mice increased plasma levels of CRP, however, in db/db mice that had higher baseline CRP levels it reduced plasma CRP level (13). Thus, it seems that exercise has more benefits in those with high basal CRP level. 

4- Age and gender effects: In children, acute and chronic exercise increases some plasma inflammatory markers such as IL-1 and TNFα which may be related to developmental changes (14). On the other hand, since female cycle has an important role in immunological response of exercise (14), in clinical studies, the age and gender of population should be considered (for example, OCP usage and menstrual stage).

5- Diet supplementation: Diet supplementation plus exercise is more effective in reducing inflammatory markers than exercise alone. Diet supplementation by omega-3 fatty acids after eccentric exercise reduced much more plasma TNFα level (15). 

## Conclusions

The effect of exercise on inflammatory markers depends on some factors such as the duration, intensity and patients’ charactristic. However, the exact role of exercise on low-grade inflammation especially non-significant changes of inflammatory markers on cardiovascular risk reduction needs further studies.
